# Preparative HPLC for large scale isolation, and salting-out assisted liquid–liquid extraction based method for HPLC–DAD determination of khat (*Catha edulis* Forsk) alkaloids

**DOI:** 10.1186/s13065-017-0337-6

**Published:** 2017-10-17

**Authors:** Minaleshewa Atlabachew, Bhagwan Singh Chandravanshi, Mesfin Redi-Abshiro

**Affiliations:** 1 Department of Chemistry, Bahir Dar University, P. O. Box 79, Bahir Dar, Ethiopia; 2 Blue Nile Water Institute, Bahir Dar University, P. O. Box 79, Bahir Dar, Ethiopia; 3 Department of Chemistry, Addis Ababa University, P. O. Box 1176, Addis Ababa, Ethiopia

**Keywords:** Khat, Alkaloids, Preparative HPLC, Salting-out assisted liquid–liquid extraction, Cathinone, Cathine, Norephedrine

## Abstract

**Background:**

Khat (*Catha edulis* Forsk) is an evergreen shrub of the *Celastraceae* family. It is widely cultivated in Yemen and East Africa, where its fresh leaves are habitually chewed for their momentary pleasures and stimulation as amphetamine-like effects. The main psychostimulant constituents of khat are the phenylpropylamino alkaloids: cathinone, cathine and norephedrine.

**Results:**

In this study, simple procedures based on preparative HPLC and salting-out assisted liquid–liquid extraction (SALLE) based methods were developed respectively for large scale isolation and the extraction of psychoactive phenylpropylamino alkaloids; cathinone, cathine and norephedrine, from khat (*Catha edulis* Forsk) chewing leaves, a stimulant and drug of abuse plant. The three khat alkaloids were directly isolated from the crude oxalate salt by preparative HPLC–DAD method with purity > 98%. In addition, a modified (SALLE) method has been developed and evaluated for the extraction efficiency of psychoactive phenylpropylamino alkaloids from khat (*Catha edulis* Forsk) chewing leaves. An in situ two steps extraction protocol was followed without dispersive SPE clean up. The method involves extraction of the samples with 1% HAc and QuEChERS salt (1.0 g of CH_3_COONa and 6.0 g of MgSO_4_) followed by subsequent in situ liquid–liquid partitioning by adding ethyl acetate and NaOH solution. The optimized method allowed recoveries of 80–86% for the three alkaloids from khat sample with relative standard deviation (RSD) values less than 15% and limits of detection (0.85–1.9 μg/mL).

**Conclusion:**

The method was found to be simple, cost-effective and provides cleaner chromatogram with good selectivity and reproducibility. The SALLE based protocol provided as good results as the conventional extraction method (ultrasonic assisted extraction followed by solid phase extraction, UAE–SPE) and hence the method can be applicable in forensic and biomedical sectors.

**Electronic supplementary material:**

The online version of this article (doi:10.1186/s13065-017-0337-6) contains supplementary material, which is available to authorized users.

## Background

Khat (*Catha edulis* Forsk) is an evergreen shrub of the *Celastraceae* family. It is widely cultivated in Yemen and East Africa, where its fresh leaves are habitually chewed for their momentary pleasures and stimulation as amphetamine-like effects. The leaves has also been introduced to western countries like Great Britain, Italy, The Netherlands, Canada, Australia, New Zealand, USA and Hungary [1–3]. Khat is usually chewed and occasionally brewed as a tea [4, 5].

**Fig. 1:**
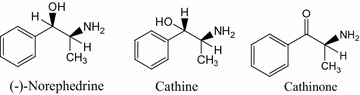
The molecular structures of the khat alkaloids

The main psychostimulant constituents/compounds of khat are the phenylpropylamino alkaloids: (−)-cathinone [(*S*)-α-aminopropiophenone], (+)-cathine [(1*S*) (2*S*)-norpseudoephedrine], and (–)-norephedrine [(1*R*) (2*S*)-norephedrine] (Fig. 1). Although there are more than 200 identified compounds in khat leaves, the phenylpropylamino alkaloids are primarily considered to be the addictive and reinforcing agents responsible for continued chewing behavior [5, 6].

Since the last 2 decades, several analytical techniques have been reported for extraction and quantitative determination of the three alkaloids. Regarding the quantification of khat alkaloids, gas chromatography–mass spectrometry (GC–MS) [6–9], gas chromatography-flame ionization detection (GC-FID) [10] and high performance liquid chromatography (HPLC) [11–15] have been reported for analyzing khat samples within pharmacological, phytochemical, forensic and law enforcement applications which were preceded by extensive sample preparation protocols after extracting the alkaloids using maceration and ultrasonication.

In natural products analysis, one of the fundamental problems arising from the complexity of the matrices is analyte extraction prior to chromatographic determination. In the determination of bioactive compounds in natural products, sample treatment is a critical step and sometimes limits the development of analytical methodologies. In this regard, for the extraction of khat alkaloids, liquid–liquid extractions (LLE) and solid phase extraction using C_18_ sorbent were repeatedly performed to clean up the interfering matrices [6–8, 10, 14, 16, 17]. However, the methods still suffered from limitations as extraction and clean-up steps were carried out separately and conditioning, washing and elution steps were time consuming during SPE clean-up.

Atlabachew and his co-workers have reported the use of matrix solid-phase dispersion for extraction and clean-up of the alkaloids from khat leaves, prior to HPLC–DAD detection [13] and molecularly imprinted polymer–solid phase extraction (MIP–SPE) [15] for the selective clean-up of khat alkaloids from aqueous extract. Despite of cleaner chromatogram obtained by the later method a lengthy procedure was needed to finalize the clean process. While the former technique seems simple and rapid, but it was found to be ineffective for eliminating co-extractives.

The salting-out assisted liquid–liquid extraction (SALLE) method is a simultaneous extraction and cleanup technique that required less time and solvent [18, 19]. The salting-out effect results in biphasic systems in mixtures composed of water and water-miscible organic solvents. In the presence of salt, the two phases can be completely distinguished in which the upper phase is mainly composed of the organic solvent [19]. It has been successfully used for the extraction and purification of a variety of chemicals, including pesticides, polycyclic aromatic hydrocarbons, antibiotics, and veterinary drugs in a wide range of matrices [20–22]. So far few papers have been reported on the use of SALLE for extraction and clean-up of natural products (isoflavones, phenolic acids and others) from plants [19, 23]. However, to the best of our knowledge, no other studies were reported to apply this technique for extraction of alkaloids from khat.

Despite several alternative clean-up protocols have been reported for the chromatographic determinations of khat alkaloids, there is still a paucity of reports describing the large scale isolation of the alkaloids from plant material. This is probably the result of challenges arising from their structural similarities and the instability of cathinone under various conditions [13, 24]. It has to be noted that the synthetic forms of these alkaloids are very expensive and are rarely accessible to researchers working on pharmacological activities of the leaves. To solve this problem isolation protocols from the cheapest natural source is a better choice but it has not been achieved except the methods reported by two group of scholars. A recent report [13] indicated that cathinone, in the form of the oxalate salt, could be obtained in high purity by acid/base extraction of the fresh uppermost young shoots of khat. However, isolation protocol for the other two alkaloids has not been reported in this paper. Schorno and Steinegger have described two isolation techniques [24]. The first involved acetylation of a mixture of the three alkaloids and the subsequent purification of cathinone acetate by preparative thin layer chromatography (TLC), while the second made use, after preparative TLC, of fractional crystallization of norephedrine and cathine from a hydrochloric acid solution. Typically, these methods result in low purities and/or poor yields. Furthermore, preparative TLC analysis is a costly and tedious process, particularly when compounds are required in significant quantities [25].

Preparative HPLC technique is particularly suitable for the isolation of a wide range of bioactive compounds, including alkaloids, from extracts of natural products [26–29] however, there are no reports describing the use of preparative HPLC for the isolation and purification of alkaloids from khat.

The aim of the present work was to optimize preparative HPLC method for the simultaneous isolation of the three khat alkaloids from khat extract; and to modify and develop the conventional SALLE extraction protocol for the analysis of psychoactive phenylpropylamino alkaloids from khat (*Catha edulis* Forsk) chewing leaves. The purpose of the modification was due to the fact that under acidic condition, the alkaloids are easily protonated and hardly partitioned into the acetonitrile phase. Whereas, under alkaline condition, the alkaloids exist in free amine base form and can be solubilized into the acetonitrile layer but phase separation cannot be achieved between the aqueous layer containing the salt and the acetonitrile phase. Thus, an alternative organic solvent was also devised.

## Experimental

### Materials and reagents

All the reagents were analytical or HPLC grade. Acetonitrile (Merck KGaA, Darmstadt, Germany), orthophosphoric acid, hydrochloric acid, diethyl ether, ethylacetate and NaOH (Merck Chemicals, Gauteng, South Africa), (−)-norephedrine [(1*R*) (2*S*)-norephedrine hydrochloride were purchased from Sigma Aldrich. (−)-Cathinone oxalate was isolated from the fresh leaves of the plant [13]. QuEChERS extraction tube and SampliQ QuEChERS AOAC Extraction kit, p/n 5982-5755 (Agilent Technologies Inc., Wilmington, DE, USA). Sodium borohydride (purity 98%) and oxalic acid (purity > 99%) were both supplied by Sigma Aldrich (Johannesburg, South Africa).

The water used was from MilliQ system from Millipore (Milford, Mass, USA). The mobile phase was filtered through a Whatman membrane filter (47 mm diameter and 2 µm pore size) while all the plant extracts were filtered through Acrodisc syringe filter (PVDF membrane with 0.45 µm pore size).

### Sample collection

Young shoots of khat (*Catha edulis* Forsk) were harvested from Bahir Dar, Ethiopia. All the samples were immediately frozen (−20 °C) to prevent decomposition of cathinone.

### Extraction of khat alkaloids for preparative HPLC based isolation

Two extraction protocols were followed for preparative HPLC based isolation.

#### Protocol 1

Khat leaves, air-dried for 5 days at ambient temperature, were powdered using a Bosch blender (Model MKM6003). The powdered sample was sieved in a nylon sieve of 100 µm. A 250 g portion of the powdered leaves was extracted using the typical acid/base extraction method for alkaloids, as reported by [13], but the volumes of solvents were adjusted. The plant material was extracted with 0.1 M HCl (3 L) by stirring with a magnetic stirrer for 90 min. The mixture was filtered using vacuum filtration. This extraction process was repeated twice, where after the combined filtrates were basified with 10% aqueous NaOH (pH 9–10). A mixture of the khat alkaloids was then extracted into diethyl ether (3 × 4 L). Oxalic acid (1% in diethyl ether) was added drop wise to the organic fraction, which was left for 24 h at 4 °C. After filtering, a 1.25 g mass was recovered as a mixture of oxalate salts (0.51% dry plant mass). The oxalate salt was directly injected to preparative HPLC.

#### Protocol 2

The oxalate salt obtained from the procedure described above was found to contain a mixture of cathinone, cathine and norephedrine. The later alkaloid, norephedrine, was much less than the other two. Thus, it was decided to convert the cathinone present to a mixture of the two diastereomers using NaBH_4_ reduction. To achieve this, excess NaBH_4_ (0.15 g in 3 mL water) was added drop wise at 0 °C, over 10 min, to 500 mg of the mixed oxalate salts dissolved in 10 mL of water. The reaction mixture was stirred for 2 h. Any residual borohydride was destroyed by the cautious addition of glacial acetic acid at 0 °C, until the solution became colorless. After basifying the mixture with 10% NaOH (pH 12–13), the alkaloids were extracted into ethyl acetate (3 × 60 mL). The combined organic layers were dried over anhydrous sodium sulfate, yielding 300 mg of a pale yellow waxy solid (0.3% dry plant mass) after removal of the solvent. Cathine and norephedrine were isolated from the products of borohydride reduction using preparative HPLC as described below.

### Preparative HPLC analysis

The isolations were performed using an Agilent 1260 series preparative HPLC (Agilent Technologies Inc., Chemetrix, South Africa), equipped with a binary pump and fitted with a Phenomenex Luna 10 u C18 column (Phenomenex; 25 cm × 10 mm × 5 μm particle size). A 10 μL of 400 mg/mL sample volume was repeatedly injected for isolation. The mobile phase consisted of aqueous phosphoric acid (0.3% v/v; pH 1.76; solvent A) and aqueous acetonitrile (10% v/v; Solvent B) at a flow rate of 9 mL/min. A linear gradient was applied from 0 to 70% solvent B in 20 min. The HPLC-diode array detector was used to monitor the individual constituents and fractions were collected following targeted peak picking method. The combined factions were evaporated to reduce the volume by half. The resulting solution was basified (pH = 10) and the particular alkaloid was extracted with diethyl ether (3 ×). Oxalic acid (1% in diethyl ether) was added dropwise to the extract and left to stand for 20 h at 4 °C to yield a white precipitate of cathinone oxalate, cathine oxalate and norephedrine oxalate, respectively.

### Determination of the purity of the isolated alkaloids

The purity of cathinone, cathine and norephedrine, isolated by preparative HPLC, was determined by analytical HPLC–DAD reported by [13].

### Extraction and clean-up method based on modified SALLE

A 0.25 g sample was weighed into a 50 mL centrifuge tube and 15 mL of 1% acetic acid (HAc) in water and kept for 15 min so as to allow the solvent to penetrate the cell wall of the plant material. QuEChERS salt kit (1.0 g of CH_3_COONa and 6.0 g of MgSO_4_) was added and vigorously shaken using vortex mixer for 6 min. Then about 2 mL of 15% aqueous NaOH solution was added to bring the solution alkaline (about pH 10, checked by universal paper indicator) followed by 10 mL of ethyl acetate was added and vigorously shaken for 2 min. The solution was kept for 1 min or centrifuged for 30 s to enhance phase separation. Exactly 5 mL of the greenish organic layer was taken using pipette, placed into 10 mL round bottomed flask and evaporated to dryness using Rota Vapor (BCHI Rotavapor R-134, Switzerland). The alkaloids were immediately re-constituted with 5.0 mL of the mobile phase (water containing 0.3% v/v phosphoric acid) while those fat soluble components were retained into the flask. The resulting solution was filtered through Acrodisc syringe filter (PVDF membrane with 0.45 µm pore size) and about 1 mL of the resulting filtered solution was placed in an auto sampler vial for HPLC–DAD analysis at 200 nm.

### Parameter optimization

To ensure method simplicity, speed, high recovery, and adequate selectivity, optimizations were made on the factors affecting these analytical requirements. Looking at the literatures for SALLE based method; usually 10–20 mL of water or 1% HAc and 10 mL of acetonitrile, acetone or ethyl acetate have been reported. Thus, having this in mind, various factors like extraction solvents (H_2_O and 1% HAc), extraction solvent volume, type of organic solvents, pH of the media, soaking time and shaking time were optimized.

### Selection of extraction solvent and solvent volume

A 0.25 g of dried and powdered khat sample was placed into 50 mL conical eppendorf tube and 10, 15, 20 and 25 mL of (water or 1% aqueous acetic acid) were added to it. The mixture was then kept for 15 min so as to allow the solvent to penetrate the cell wall of the plant material. Then, the QuEChERS salt (1.0 g of CH_3_COONa and 6.0 g of MgSO_4_) was added to the mixture and shaken vigorously for 6 min on a vortex mixer. To the extract, 1–2 mL of 15% NaOH was added to it to bring the pH to 10. Subsequently, 10 mL of ethyl acetate was added and shaken for 1 min. Finally 5 mL of the supernatant was taken after centrifugation of the mixture for 30 s. The organic layer was removed and reconstituted with the mobile phase. The extraction was performed in duplicate.

### Optimization of soaking and shaking time

Before adding the QuEChERS salt, the sample was soaked into the extraction solvent (1% HAc) so as to allow the solvent to swollen the cell wall of the plant material and facilitate the release of analytes from the matrix into the solution. Thus, four soaking conditions (0, 5, 10 and 20 min) were selected while the remaining procedures were the same as above. Duplicate analysis was carried out.

### Optimization of shaking/extraction time

Shaking the mixture after soaking the sample and adding the QuEChERS salt is critical stage where by the alkaloids are expected to be released from the matrix into the aqueous phase. Taking into account that the alkaloids were present in their natural form in the samples analyzed, we tested whether the increase in the shaking time might increase the efficiency of extraction. Thus, portions of 0.25 g of powdered sample were taken and placed into 50 mL of centrifuged tube containing 15 mL of 1% HAc. After soaking the mixture for 15 min, QuEChERS salt was added and shaken vigorously for the set periods of time (2, 4, 6 or 10 min). Then the rest of the procedures were followed as above. The experiment was performed in duplicate.

### Effect of pH

Once parameters like solvent volume (15 mL), soaking time (15 min) and shaking time (6 min) were optimized using ethyl acetate as an organic solvent, pH of the media was evaluated for quantitative extraction of the alkaloids. Most of the reported SALLE based protocols were based on extraction of the substances in acidic media. In this study, however, acidic condition stabilizes the alkaloids in the aqueous phase due to protonation of the amine nitrogen and hence will not be solubilized in the organic phase. Thus 15% NaOH was added to make the solution alkaline and enhance solubility of the alkaloids in the organic phase. Three different pH conditions (8, 10 and 12) were investigated to select a pH-value that could be adequate for the quantitative extraction of the analytes from the aqueous phase into the organic layer. The extraction was performed in duplicate.

### Effect of salt addition on the extraction yield of the alkaloids

In the SALLE methodology, phase separation was induced by the addition of various salts—avoiding the use of potentially toxic and expensive co-solvents. The salt most commonly used is MgSO_4_, which reduces the volume of the aqueous phase and facilitates the partitioning of polar analytes into the organic phase [30]. In order to evaluate the significance of the salt on the extraction efficiency and phase separation, a duplicate extraction was conducted following the same procedure as above without the addition of the salt, i.e. 0.25 g of sample was soaked into 15 mL of water for 10 min and then vigorously shaken for 6 min. After adjusting the pH to 10 an organic solvent was added and shaken for 2 min.

### Ultrasonic assisted extraction followed by SPE (UAE–SPE)

For the UAE–SPE experiments, the procedure developed by [11] was used. Namely, 0.25 g of sample extracted 3 times with a total of 50 mL 0.1 N HCl in ultrasonic bath for 45 min. The combined filtrate was evaporated to dryness at 40 °C using vacuum rotary evaporator. The residue was dissolved in the mobile phase and passed through a pre-conditioned SPE cartridge. Then the cartridge was eluted with the mobile phase. The extraction was performed in duplicate.

### HPLC analysis of the extracts

The analyses were performed using an Agilent 1200 Series HPLC (Agilent Technologies Inc., Chemetrix, South Africa), equipped with a binary pump and fitted with an Ascentis™ C8 column (Supelco; 25 cm × 4.6 mm × 5 μm particle size). A 5.00 μL sample volume was analysed throughout. The diode array detector was used for quantification at 200 nm. The mobile phase consisted of aqueous phosphoric acid (0.3% v/v; pH 1.76; solvent B) and aqueous acetonitrile (15% v/v; solvent A) at a flow rate of 1.5 mL/min in a gradient profile as follows: 0–5 min (95–92% B in A, linear gradient); 5–12 min (92–60% B in A, linear gradient). For the UAE–SPE extract, the column was further eluted with (60–10% B in A, linear gradient) from 12–20 min. Then the condition was reversed to its initial condition. For each duplicate extraction, duplicate HPLC analysis was done (n = 4).

### Reproducibility and recovery

The reproducibility of the analytical methods and the repeatability of the extraction procedure were assessed by evaluating the peak area ratio variation of the three alkaloids present in the extracts. Two replicates were performed for each extraction assay and two replicate HPLC–DAD analyses were performed on each filtrate. The recovery of the SALLE was assessed by measuring the recovery of the spiked concentrations of 50, 80 and 80 µg/mL of norephedrine, cathine and cathinone, respectively, in a sample containing to 0.25 g of khat after passing all the processes mentioned above.

## Results and discussion

### Isolation of the three khat alkaloids from the oxalate salt of crude extract

**Fig. 2:**
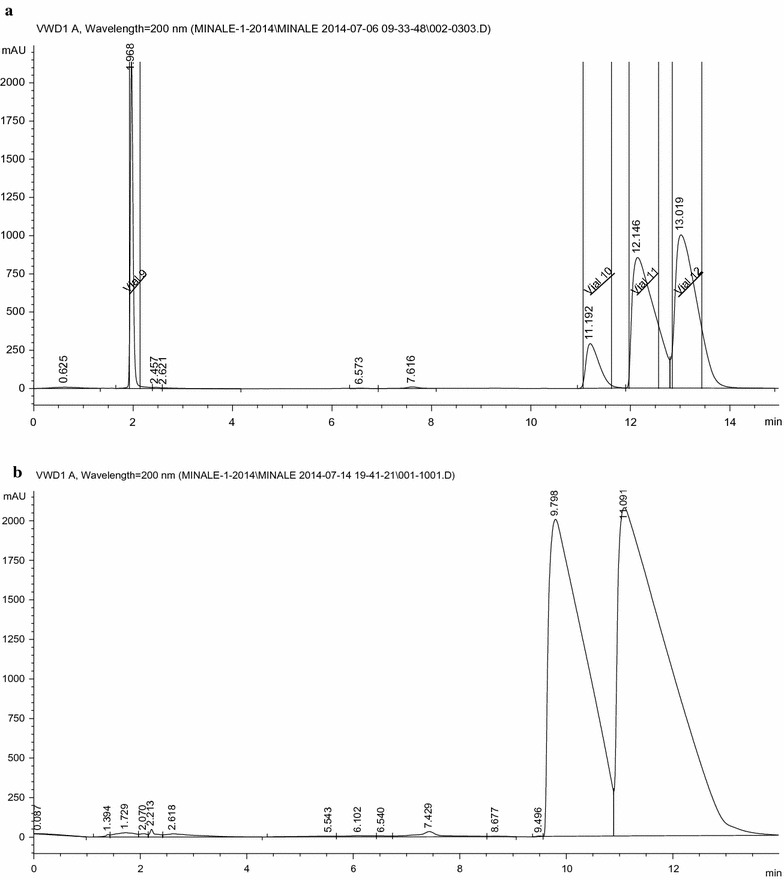
Preparative HPLC chromatogram of the **a** oxalate salt (right to left: cathinone, cathine, norephedrine and oxalate ion) and **b** borohydride reduced product (right to left: cathine and norephedrine)

**Fig. 3:**
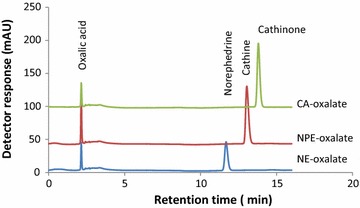
Superimposed HPLC–DAD chromatograms of fractions collected from prep HPLC and concentrated as oxalate salts. *CA* cathinone, *NPE* norpseudoephedrine or cathine, *NE* norephedrine

Originally the concentrated aqueous extract was supposed to be used for the preparative HPLC–DAD isolation protocol. But due to the following reasons, the oxalate salt was preferred: (1) in the aqueous crude extract, there were several compounds eluting with the analytes of interests and hence the automatic fraction collector was forced to collect several fractions per a single injection and hence needs replacement of empty fraction collector vials in every one or two injections. (2) After every injection, post run analysis with pure acetonitrile mobile phase followed by column equilibration with the initial mobile phase flow conditions was required to elute strongly interacting compounds in the column; otherwise there were a chance of co-eluting compounds with the analyte of interest during the subsequent injections. This post run and equilibration step is time consuming and needs several milliliters of the mobile phase. (3) when crude extract is injected only small traces of the alkaloids are isolated per injection volume due to the fact that the composition of other constituents are significantly higher than the alkaloids do. Thus, we decided to partially purify the alkaloids via precipitation with oxalic acid so that the aforementioned problems have been minimized. Figure 2a shows the chromatogram obtained from the preparative HPLC for the oxalate salt extract. Looking at the chromatogram, the concentration of norephedrine was small compared to the other two. Then we decided to reduce cathinone to cathine and norephedrine using sodium borohydride. As a result significant amount of norephedrine and cathine were isolated from a single injection. Figure 2b shows the chromatogram obtained from prep. HPLC for borohydride reduced product. The similar fractioned were combined together, applied LLE and treated as oxalic acid as above. Each alkaloid was recovered as oxalate salt. Figure 3 shows the overlaid chromatograms of the individual fractions as oxalate salt from prep HPLC. Looking at the figure, almost pure alkaloids have been obtained from this experiment. Therefore, liquid–liquid extraction followed by preparative HPLC could be used to isolate reasonably pure khat alkaloids.

### Results for salting-out assisted liquid–liquid extraction (SALLE) method

#### Sample comminuting

The mechanical force generated during vortex mixing the mixture and the exothermic heat produced during the hydrolysis of the salt added is responsible for the extraction of the alkaloids from the matrix into the aqueous phase. But these mechanisms of extraction seem to be lower compared with other extraction approaches. It is utmost important to ensure that the sample is powdered to fine particles to maximize the surface area and ensure better extraction efficiency. Thus, the ground sample was sieved in nylon sieve of 100 µm before extraction.

#### Selection of extraction solvent

In order to quantitatively liberate the analytes from the matrix and subsequently enrich them into the organic phase, optimum solvent type and its volume was deemed important. In the literature both water [15], acidified water [11–14, 16, 17] and methanol [10] were used to extract khat alkaloids using ultrasonic assisted extraction, maceration and others. Secondly, in QueChERS and salting out assisted liquid–liquid extraction, these two solvents have also been reported as extraction solvents together with acetonitrile [19–23]. It was clearly stated that acidic condition could stabilize cathinone during extraction hence most authors argue that acid condition is more preferable to extract khat alkaloids [14]. Thus, in this study, it was aimed to see the effect of acidic condition (1% v/v acetic acid in water) on extraction efficiency of the alkaloids as compared to pure water. However, it has to be noted that highly acid condition requires more basic solution for neutralization during partitioning of the analytes in the organic phase. Hence higher concentrations of acetic acid were not used in the present study. Results of the analysis are shown in Additional file 1: Figure S1.

Looking at Additional file 1: Figure S1, both water and 1% HAc were found to successfully extract the three alkaloids from the plant material under identical conditions. However, 1% HAc was found to be more efficient as compared with pure water. This result is corroborating with earlier reports on other extraction protocols like matrix solid phase dispersion and ultrasonic assisted extraction [13].

Looking at the effect of extraction solvent volume on the extraction yield, it has been noticed that increase in solvent volume (up to 20 mL) caused slight increment in the extraction yield of the alkaloids. But further increase in solvent volume yielded lower concentration of the alkaloids when both of the solvents were considered. This might be due to lowering of the heat generated in the system as a result of exothermic reaction between water and the added salt which is supposed to be sufficient to liberate the alkaloids from the cell wall of the plant material in addition to the mechanical force applied during vortex mixing of the mixtures. Therefore, 15 mL of 1% HAc was selected as optimum solvent and solvent volume for this study.

#### Optimization of soaking and shaking time

**Fig. 4:**
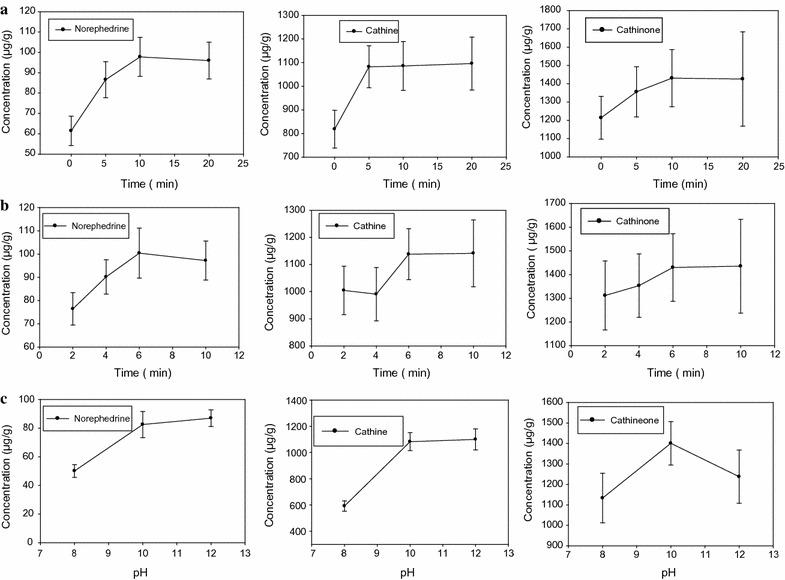
**a** Effect of soaking time, **b** effect of shaking time and **c** effect of pH on the extraction efficiency of norephedrine, cathine and cathinone

Since the matrix analyte interaction is much stronger in case of natural products, it is absolutely important to soak the sample in the extraction solvent so as to give more room to the solvent to penetrate the cell wall of the plant material and swollen it to ensure better extraction efficiency of the alkaloids. Results of the analysis are shown in Fig. 4a.

Even though, soaking of the sample for 20 min gave better result, the difference in yield with the 10 min soaking time was not significant. So, soaking of the sample between 10 and 20 min can be recommended as optimum time for soaking the samples. Thus 15 min was selected as optimum soaking time for the study. The effect of prolonged soaking time (45 min and over night) was also studied. However, the effect was insignificant.

As it has been mentioned above, shaking the mixture for a set period of time is a critical step to enhance the extraction efficiency of the alkaloids. As it can be seen from Fig. 4b, when the shaking/extraction time increased, a rise in the analytical signal was observed and hence 6 min was considered as efficient time for shaking the soaked sample.

#### Effect of pH

The pH of the extraction must be controlled. Unlike the conventional SALLE, extraction of the alkaloids was carried out in alkaline solution since alkaloids are easily partitioned into the organic phase when the solution is basic. Thus, selection of optimum pH is mandatory so as to ensure quantitative recovery of the alkaloids in the organic phase. Figure 4c shows the effect of pH on the extraction yield of the alkaloids. As it can be noted from the figure, pH 10 was found to yield significantly better analytical signal than pH 8 and 12. Therefore, it was regarded as optimum pH for the study.

#### Evaluation of the analytical method

**Fig. 5:**
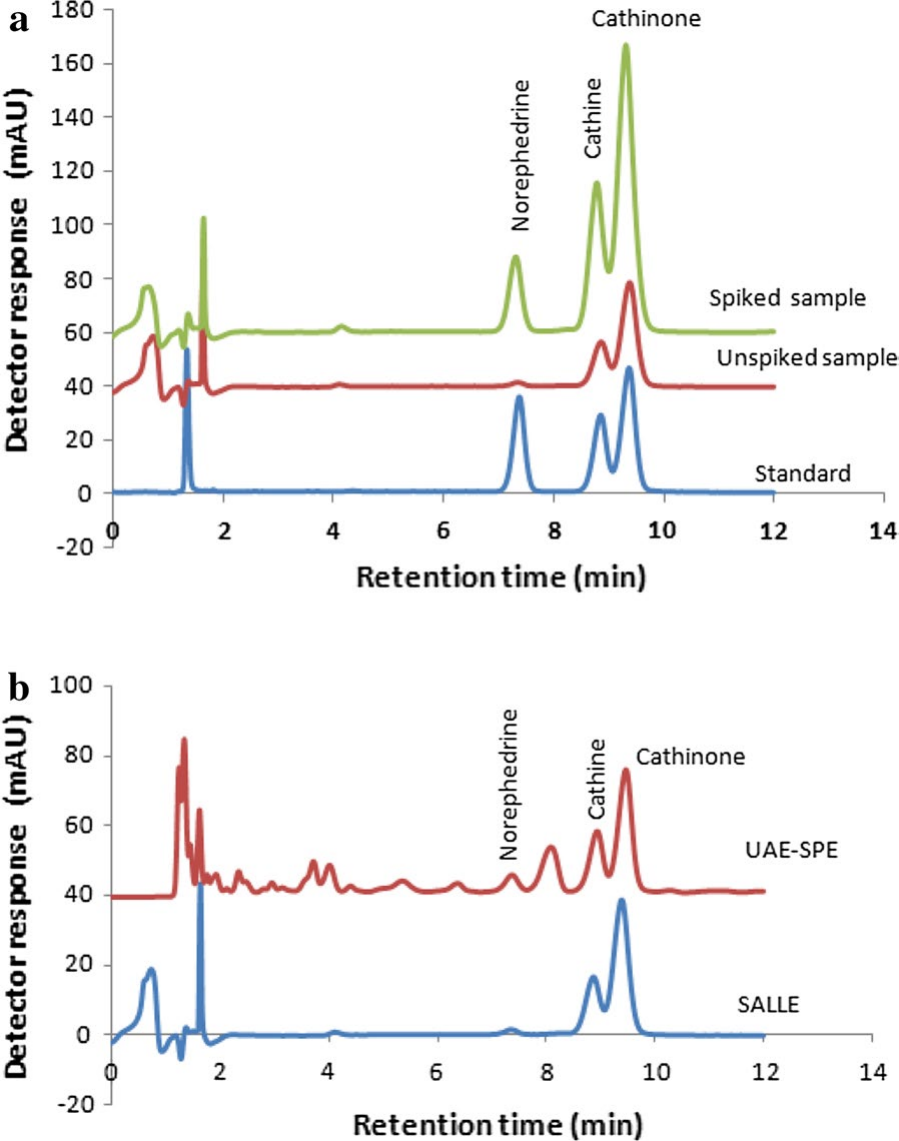
Superimposed HPLC–DAD chromatograms obtained for: **a** spiked and unspiked samples extracted with SALLE, and pure standards (60, 60, and 30 µg/mL respectively of norephedrine, cathine and cathinone) and **b** sample extracted with SALLE and UAE–SPE methods

Once the SALLE parameters were optimized, known concentrations of the alkaloids (50, 80 and 80 µg/mL of norephedrine, cathine, and cathinone) were spiked to the khat sample (containing 2.4, 30.2, and 39 µg/mL of norephedrine, cathine, and cathinone) and extraction was conducted. The concentrations of the three alkaloids in the spiked samples were found to be 42.5, 96.3, and 107.8 µg/mL, respectively. Figure 5a shows the chromatograms of the SALLE extracts of (1) unspiked khat sample, (2) the same khat spiked with norephedrine, cathine and cathinone and (3) standards. Results of the analysis showed that all the compounds were extracted efficiently and displayed good recoveries (80–86%) with % RSD values ranging from (11 to 13% for n = 4 runs). The limit of detection (LOD) of the method was calculated using the calibration curve parameters after the linear calibration curves were produced by plotting the analyte peak area against the corresponding concentrations of the alkaloids. A good linearity response greater than 0.999 was obtained for the three analytes in the concentration range of 1.5–240, 1.5–240 and 0.75–120 µg/mL for norephedrine, cathine, and cathinone, respectively. The slope and intercept values for calibration curves were y = 10.81x − 16.5 (R^2^ = 0.9995) for norephedrine, y = 10.5x − 9.1 (R^2^ = 0.9991) for cathine and y = 20.1x − 12.8 (R^2^ = 0.9998) for cathinone. The LOD was established using LOD = 3.3(s/S), where s is the standard deviation of the intercept and S is the slope of the curve. The LOD obtained for norephedrine, cathine and cathinone were 1.93, 1.56 and 0.85 µg/mL, respectively.

#### Comparison of SALLE method with ultrasonic assisted extraction followed by SPE

The SALLE method was compared with ultrasonic assisted extraction followed by SPE (UAE–SPE). Results are shown in Table 1 and Fig. 5b. From the figure, it can be seen that a cleaner chromatogram was obtained in the case of SALLE when compared with UAE–SPE. From the table, it was observed that comparable yield of khat alkaloids (cathine and cathinone) could be obtained when SALLE was applied as compared to UAE–SPE. But significantly lower concentration of norephedrine was noticed on the other hand than did the UAE–SPE (Table 1). In addition, the precision of SALLE based protocol was lower for cathinone than that of UAE–SPE. However, SALLE method is much easier, faster and more than four samples/aliquots can be handled at a time. Furthermore, a cleaner chromatogram could be obtained compared with UAE/SPE.

**Table 1:** Comparison of the precision and extraction efficiency of SALLE with UAE–SPE

Protocol	Norephedrine^a^ (µg/g)	% RSD	Cathine^a^ (µg/g)	% RSD	Cathinone^a^ (µg/g)	% RSD
SALLE	96 ± 9.1	9.5	1210 ± 120	9.9	1560 ± 190	12.2
UAE–SPE	170 ± 12	7.6	1290 ± 68	5.3	1400 ± 94	6.71

^a^Values are represented by mean ± SD (n = 4); *RSD* relative standard deviation

## Conclusion

In this report, a semi-preparative HLPC and modified SALLE based methods were optimized for the simultaneous isolation and for the HLPC-DAD determination of khat alkaloids, respectively. Due to the complexity of the khat extract, partial purification of the crude extract following liquid–liquid extraction and precipitation with oxalic acid, made possible the isolation of each of the alkaloids using preparative HPLC by injecting 10 µL of 400 mg/mL of the crude oxalate salt. Per a single injection it was possible to isolate about a milligram of each analyte as oxalate salt. Norephedrine is naturally found at lower concentration compared to the other two alkaloids. Thus treating the crude oxalate salt by NaBH_4_ converts cathinone to cathine and norephedrine. This reduction procedure allows large scale isolation of norephedrine and cathine per single run with high purity.

In addition, SALLE based method was developed, optimized and evaluated for the extraction of naturally present alkaloids from khat (*Catha edulis* Forsk) chewing leaves samples. The method follows two extraction steps where the analytes are first extracted into the aqueous phase followed by partitioning into the organic phase after pH adjustment was carried out. The results showed that the method was satisfactory in terms of selectivity and reproducibility with a cleaner chromatogram without any clean up step. It was simple, cost-effective, and can be used as a useful analytical extraction method to measure the khat alkaloids concentration in forensic and biomedical investigations.

## Additional file

**Additional file 1: Figure S1.** Optimization of volume of 1% HAc and H2O for extraction of (a) norpseudoephedrine (NPE) or cathine, (b) norephedrine (NE) and (c) cathinone (CA).
