# Supplementary material for: Preparative HPLC for large scale isolation, and salting-out assisted liquid–liquid extraction based method for HPLC–DAD determination of khat (Catha edulis Forsk) alkaloids
Source: Chem Cent J. 2017 Oct 17;11:107. doi: 10.1186/s13065-017-0337-6 (PMC5645267; doi:10.1186/s13065-017-0337-6)
Supplement: Supplementary file 1 — Additional file 1: Figure S1. Optimization of volume of 1% HAc and H2O for extraction of (a) norpseudoephedrine (NPE) or cathine, (b) norephedrine (NE) and (c) cathinone (CA). [file 13065_2017_337_MOESM1_ESM.docx]

**Additional file 1**

**Figure S1.** Optimization of volume of 1% HAc and H_2_O for extraction of (a) norpseudoephedrine (NPE) or cathine, (b) norphedrine (NE) and (c) cathinone (CA)
